# Bacterial Extracellular DNA Promotes β-Amyloid Aggregation

**DOI:** 10.3390/microorganisms9061301

**Published:** 2021-06-15

**Authors:** George Tetz, Victor Tetz

**Affiliations:** Department of Neuroscience, Human Microbiology Institute, New York, NY 10128, USA; g.tetz@hmi-us.com

**Keywords:** Alzheimer’s disease, amyloid-beta, amyloid plaques, bacterial DNA, protein aggregation

## Abstract

Alzheimer’s disease is associated with prion-like aggregation of the amyloid β (Aβ) peptide and the subsequent accumulation of misfolded neurotoxic aggregates in the brain. Therefore, it is critical to clearly identify the factors that trigger the cascade of Aβ misfolding and aggregation. Numerous studies have pointed out the association between microorganisms and their virulence factors and Alzheimer’s disease; however, their exact mechanisms of action remain unclear. Recently, we discovered a new pathogenic role of bacterial extracellular DNA, triggering the formation of misfolded Tau aggregates. In this study, we investigated the possible role of DNA extracted from different bacterial and eukaryotic cells in triggering Aβ aggregation in vitro. Interestingly, we found that the extracellular DNA of some, but not all, bacteria is an effective trigger of Aβ aggregation. Furthermore, the acceleration of Aβ nucleation and elongation can vary based on the concentration of the bacterial DNA and the bacterial strain from which this DNA had originated. Our findings suggest that bacterial extracellular DNA might play a previously overlooked role in the Aβ protein misfolding associated with Alzheimer’s disease pathogenesis. Moreover, it highlights a new mechanism of how distantly localized bacteria can remotely contribute to protein misfolding and diseases associated with this process. These findings might lead to the use of bacterial DNA as a novel therapeutic target for the prevention and treatment of Alzheimer’s disease.

## 1. Introduction

The misfolding, aggregation, and accumulation of amyloid-forming proteins are key pathological occurrences in protein misfolding diseases [1] These diseases include systemic amyloidosis, type 2 diabetes, and a broad variety of neurodegenerative diseases, such as Alzheimer’s disease (AD), Parkinson’s disease, Huntington’s disease, amyotrophic lateral sclerosis, and many others [2,3,4,5]. AD is the most common cause of dementia worldwide and is a leading cause of death in elderly individuals in the developed world [6]. The disease is characterized by a progressive cognitive decline and amnestic impairment. This is due to specific neuropathological changes in the brain as a result of the formation and accumulation of neurotoxic extracellular amyloid plaques and intracellular neurofibrillary tangles [7,8]. The formation and deposition of amyloid plaques appear to be among the earliest and most significant pathological events in AD [9]. These amyloid plaques are composed of aggregated amyloid-β protein (Aβ), which is a 42-residue peptide formed from the enzymatic processing of the amyloid precursor protein [10,11,12,13]. A seeding nucleation prion-like mechanism is responsible for a conformational switch in the Aβ protein, which results in the misfolding, self-propagation, and fibrillar aggregation of Aβ, which becomes deposited in the brain parenchyma in the form of neuritic amyloid plaques [1]. However, despite decades of research, the mechanisms and factors responsible for the initiation of Aβ misfolding remain elusive [14,15]. 

It is believed that the interaction between genetic and environmental factors contributes to AD development [16,17]. Many proteins and non-protein components have been shown to promote or inhibit Aβ misfolding and aggregation in vitro and in vivo; however, whether they are involved in the AD process remains unclear [18,19]. The lack of a clear understanding of the factors that trigger the cascade of AD might in part be responsible for the numerous failures of anti-AD drugs in clinical trials that have occurred in recent years [20].

Recently, an increasing number of studies have implicated microorganisms and viruses as culprits in AD and other neurodegenerative diseases [21,22,23,24,25,26]. At the same time, their exact mechanisms through which they trigger AD remain unclear. The dominant theory is that changes in microbiota can induce neuroinflammation or alter metabolic, endocrinal, and immunological pathways [18]. The involvement of microorganisms is believed to occur both directly, via their migration to the brain followed by the activation of host microglia and peripheral immune cells [27], or indirectly, through regulation of the microbiota–gut–brain axis and the passage of bacterial components and virulence factors from the impaired blood–brain barrier to the CNS [28,29,30,31]. 

We have recently shown another possible universal pathway by which microorganisms might trigger diseases associated with protein misfolding. We discovered that bacterial extracellular DNA (eDNA) leads to the modification of already synthesized proteins that are converted into altered heat-resistant Tetz-proteins or they aggregate into cross-β structures [32,33]. We found that the DNA released from bacteria associated with Parkinson’s disease, type 1 diabetes, and AD can trigger α-synuclein, islet amyloid polypeptide, and tau misfolding, thus implicating it as a potential virulence factor in these pathologies [33,34,35]. These data are aligned with our previous findings, showing that the deoxyribonuclease I enzyme that cleaves extracellular DNA might have some benefit for patients with AD [36].

To test whether DNA triggers Aβ misfolding, we used eDNA from bacterial species known to be associated with AD, as well as human DNA. We used DNA from the oral bacteria Porphyromonas gingivalis, Tetzerela hominis, Escherichia coli, and Borrelia burgdorferi, which has been detected in the cerebrospinal fluid (CSF) and postmortem brains of individuals with AD or have been suggested to be AD-discriminative microorganisms [27,37,38,39]. We hypothesized that eDNA from these representative oral microbiota might be specifically implicated in AD development through the mouth–brain axis. Indeed, DNA of oral bacteria can reach the CNS, not only through the typical means of the weakened blood–brain barrier [18,40], but also through direct release by these microorganisms into the brain following their invasion of the CNS due to neurotropism and spread through cranial nerves [27,41,42,43]. In this study, using an in vitro Aβ aggregation assay, we observed that eDNA of some bacteria could induce Aβ misfolding and aggregation.

## 2. Materials and Methods 

### 2.1. Sources and Procedures for DNA Extraction

Extracellular DNA was extracted from the matrix of *Escherichia coli* ATCC25922, *E. coli* ATCC 472217, *E. coli* G39, *E. coli* dPHF, *E. coli* MUP6, *Porphyromonas gingivalis* ATCC BAA-308, *Burkholderia burgdorferi* ATCC35210, and *Tetzosporium hominimis* VT-49. All bacterial strains were subcultured from freezer stocks onto Columbia agar plates (Oxoid, Hampshire, UK) and were incubated at 37 °C for 48 h. Human genomic DNA (0.2 g/L in 10 mM tris-HCl, 1 mM EDTA, pH 8.0, Cat. No. 11691112001) was purchased from Sigma (Sigma-Aldrich, St Louis, MO, USA) and consisted of high molecular weight (>50,000 bp) genomic DNA isolated from human blood.

### 2.2. DNA Fragment Size Characterization with an Agilent Bioanalyzer

The sizes of DNA from different *E. coli* strains were analyzed using an Agilent Bioanalyzer 2100 instrument (Agilent Technologies, Santa Clara, CA, USA) following the manufacturer’s recommended protocol using Agilent 2100 Expert software.

### 2.3. DNA and RNA Extraction and Purification

To isolate extracellular nucleic acids, the cell suspension was centrifuged and separated from the extracellular matrix by washing twice in phosphate-buffered saline (PBS; pH 7.2; Sigma, St Louis, MO, USA) and centrifuged at 4000× *g* for 15 min (Microfuge 20R, Beckman Coulter, Brea, CA, USA) followed by resuspension in PBS. Next, the supernatant was filtered through a 0.22 µM filter to remove any bacterial cells. Extracellular DNA was extracted using a DNeasy Blood & Tissue Kit (Qiagen, Hilden, Germany) according to the manufacturer’s instructions. Purified DNA quality was assessed using a NanoDrop OneC spectrophotometer (Thermo Fisher Scientific, Waltham, MA, USA). Samples with a DNA OD 260/280 = 1.8 − 2.0 were used for further analysis.

Extracellular or total intracellular RNA was purified using the RNeasy Mini Kit (Qiagen) according to the manufacturer’s recommended protocol. The evaluation of RNA quantity and quality was performed spectrophotometrically based on UV absorbance at 230/260/280 nm with a NanoDrop OneC spectrophotometer (Thermo Fisher Scientific, Waltham, MA, USA). Some of the DNA probes were treated with 100 units of DNase I (Sigma-Aldrich, St Louis, MO, USA) for 20 min at 37 °C to degrade DNA in the probes.

### 2.4. Aβ Synthesis and Preparation 

Aβ1-42 peptide was produced by solid-phase synthesis at The ERI Amyloid Laboratory, LLC, and purified (>95% purity) by reverse-phase chromatography. Peptides were dissolved in 50% acetonitrile, frozen, and lyophilized overnight. Lyophilized Aβ was dissolved in 10 mM NaOH (pH 12). The material was centrifuged in a 30 kDa cut-off filter for 12 min at 14,000× *g* at 4 °C to obtain the “seed-free” solution. 

### 2.5. In Vitro Aggregation Assay and Half-Time Analysis 

Aβ seed-free solution was diluted to a final concentration of 6 µM in 50 mM tris-HCl and incubated with intermittent shaking (450 rpm) at 15 °C. DNA probes were added in water, and the same volume of water was used as negative controls. Then, 5 µM thioflavin T (ThT) was added so that aggregation over time could be measured by fluorescence at 485 nm after 435 nm excitation. The maximum fluorescence intensity of a sample was used to calculate the normalized intensity for presentation purposes. Thus, we normalized ThT fluorescence taken at each timepoint to 100% of the maximum value. To quantify the kinetics of Aβ aggregation, we determined the half-time (t1/2), at which normalized ThT fluorescence reaches half the maximum intensity [44].

### 2.6. Statistical Analysis

The significance of the differences in aggregation kinetics of Aβ in the presence of different DNA samples was analyzed by one-way ANOVA, followed by Tukey’s multiple comparison post-test. To compare the effect of different DNA samples, we estimated alterations of ThT fluorescence and the t1/2, which corresponds to the time at which 50% aggregation is obtained. The level of significance was set at *p* < 0.05.

## 3. Results

Effect of DNA from Different Organisms on Aβ1-42 Aggregation Kinetics

**Figure 1:**
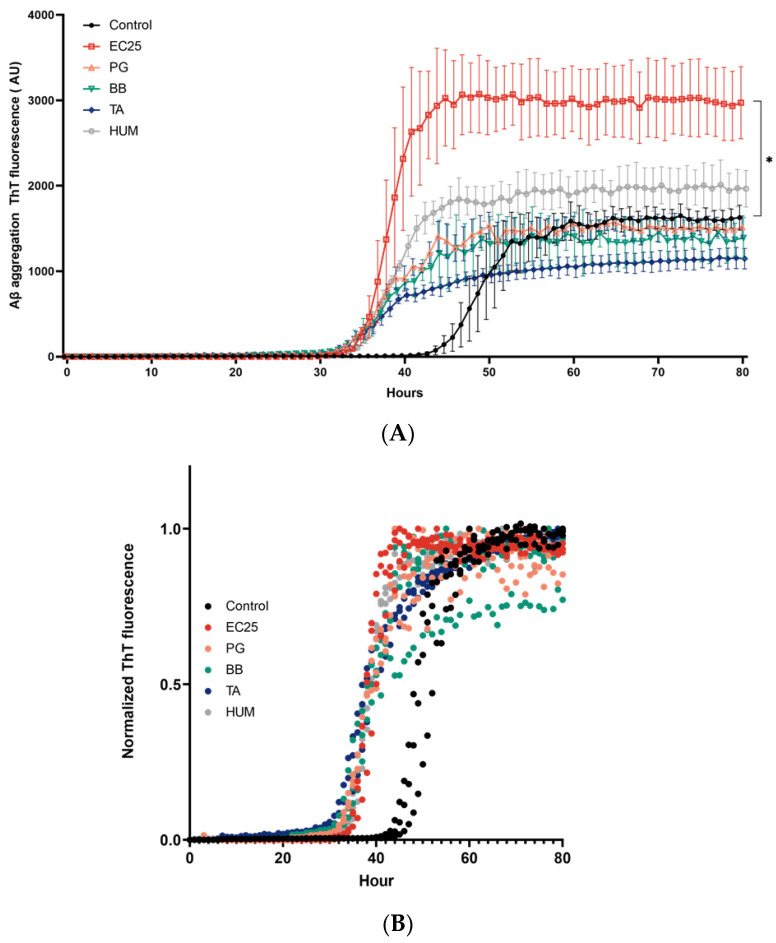

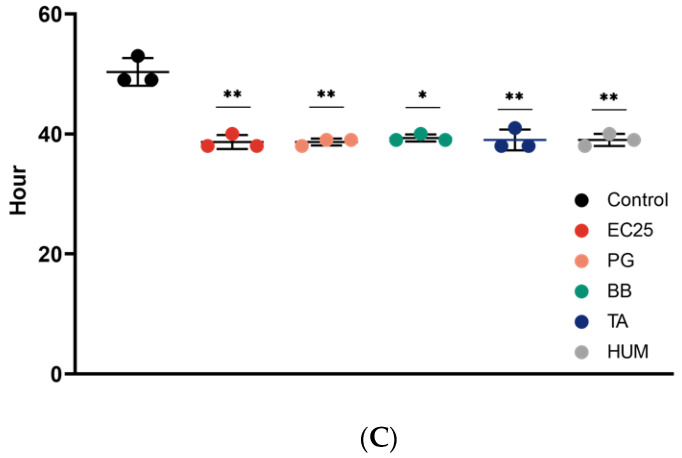
Effects of bacterial and human DNA on amyloid β (Aβ) aggregation. ThT fluorescence assay was used to monitor the aggregation of Aβ (1-42) at 6 µM in 50 mM tris-HCl in the presence and absence of bacterial DNA of *E. coli* ATCC25922 (EC25), *P. gingivalis* (PG), *Borrelia burgdorferi* (BB), *T. alzheimeri* (TA), or human DNA (HUM) at 1000 ng/mL. (**A**) ThT fluorescence as a function of time (h), (**B**) normalized ThT fluorescence as a function of time, (**C**) and the corresponding t1/2 values. Each experiment was performed in triplicate. For images (**A**) and (**C**), data are presented as mean ± SD * *p* ≤ 0.05 ** *p* ≤ 0.01; for (**B**), the individual traces from three replicates are shown.

To investigate the effect of DNA on Aβ misfolding and aggregation, we studied the fibrillation kinetics of Aβ in the absence or the presence of extracellular DNA extracted from different bacterial species that were previously shown to be associated with AD, as well as human cells [27,37,38,39]. As it is well known that the onset of Aβ aggregation varies significantly within different experiments and replicates of the same sample, all experiments were performed at the same time with the same reagents, and ThT fluorescence curves were normalized. First, we found that DNA of different organisms taken at a fixed concentration of 1000 ng/mL affected Aβ aggregation differently. The maximum fluorescence value was considerably higher only following the addition of DNA from *E. coli* ATCC 25922 (Figure 1A). No significant effect on maximum ThT fluorescence was detectable after the addition of eDNA from *P. gingivalis* ATCC BAA-308, B. burgdorferi ATCC 35210, or *T. hominimis* VT-49 gen. nov, sp. nov, a new species isolated from the oral cavity of a patient with AD, as well as human DNA. 

To allow for an easier visual comparison of the kinetics of protein aggregation, we normalized fibrillation curves (Figure 1B). It was clearly seen that in both control and DNA-treated probes, Aβ (1-42) aggregation exhibits a characteristic sigmoidal curve indicative of amyloid formation via primary nucleation. We observed shortening of the lag phase, defined as the time at which aggregation begins following the addition of all DNA samples used [44]. Aβ seeded with bacterial or human DNA showed a lag phase of approximately 10 h before that of the untreated control probe group (*p* < 0.05). As expected, we also found a reduction in the t1/2 values of the amyloid growth phase after the addition of any DNA probes (Figure 1C). 

**Figure 2:**
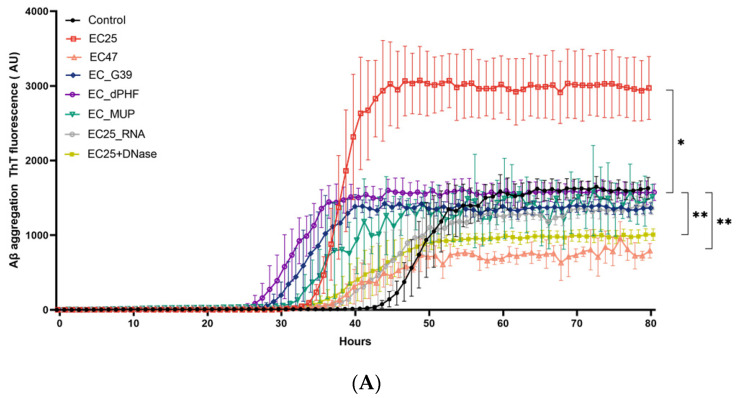

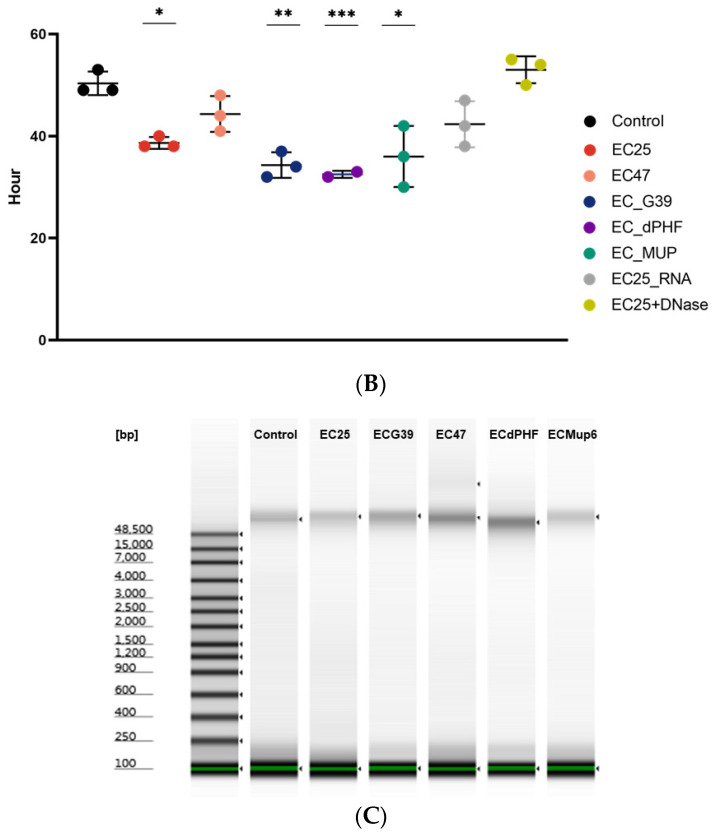
Effect of eDNA and RNA of different Escherichia coli strains on amyloid β (Aβ) aggregation. To study the effect of DNA on Aβ aggregation, Aβ1-42 oligomers were incubated with preparations containing 1000 ng of nucleic acids extracted from different strains of *E. coli*. eDNA of *E. coli* ATCC29522 (EC25), *E. coli* ATCC472217 (EC47), *E. coli* G39 (ECG39), *E. coli* dPHF (ECdPHF), and *E. coli* MUP6 (ECMup6), RNA of *E. coli* ATCC29522 (EC25_RNA), and eDNA of *E. coli* ATCC29522 treated with DNase (EC25+DNase) were used. (**A**). ThT fluorescence as a function of time in the presence of indicated DNA and RNA probes (h); (**B**) t1/2 values of aggregation. Values represent the mean ± SD of experiments performed in triplicate. *** *p* ≤ 0.001, ** *p* ≤ 0.01 * *p* ≤ 0.05. (**C**) Bioanalyzer gel image for eDNA extracted from different strains of *E. coli*.

As the eDNA of *E. coli* ATCC25922 was shown to be the only DNA sample to trigger higher ThT fluorescence values, we next tested the effect of eDNA from different *E. coli* strains on this process (Figure 2A). Along with two ATCC strains, *E. coli* ATCC25922 and *E. coli* ATCC472217, we used three *E. coli* clinical isolates, G39, dPHF, and MUP6, which were isolated from the oral cavity of patients with advanced AD. Upon addition of eDNA of different *E. coli* strains, we found that the ThT signal was only increased by the eDNA of *E. coli* ATCC25922 (*p* < 0.01). It was slightly inhibited by eDNA of *E. coli* ATCC 472217 and was mostly unaffected by the addition of eDNA from other *E. coli* (Figure 2A). At the same time, we found that eDNA of all *E. coli* strains except *E. coli* ATCC 472217 significantly shortened the lag-phase and corresponding t1/2 values (Figure 2A,B). 

Surprisingly, we observed aggregation curves with the shortest lag phase and the most decreased t1/2 following the adding of DNA of E. coli dPHF-; however, it did not increase ThT fluorescence. 

As expected, the destruction of eDNA from *E. coli* ATCC25922 by DNase I and its subsequent addition to Aβ (1-42) completely inhibited the increase in ThT fluorescence and decrease in the t1/2, which were observed for the undigested t1/2 (Figure 2A,B). This is related to the specificity of the eDNA and not to the effect of the seeding of beta-amyloid misfolding by nucleotides. We also determined that RNA from *E. coli* ATCC25922 had not increased ThT fluorescence or accelerated the kinetics of t1/2 for Aβ aggregation (Figure 2B). 

**Figure 3:**
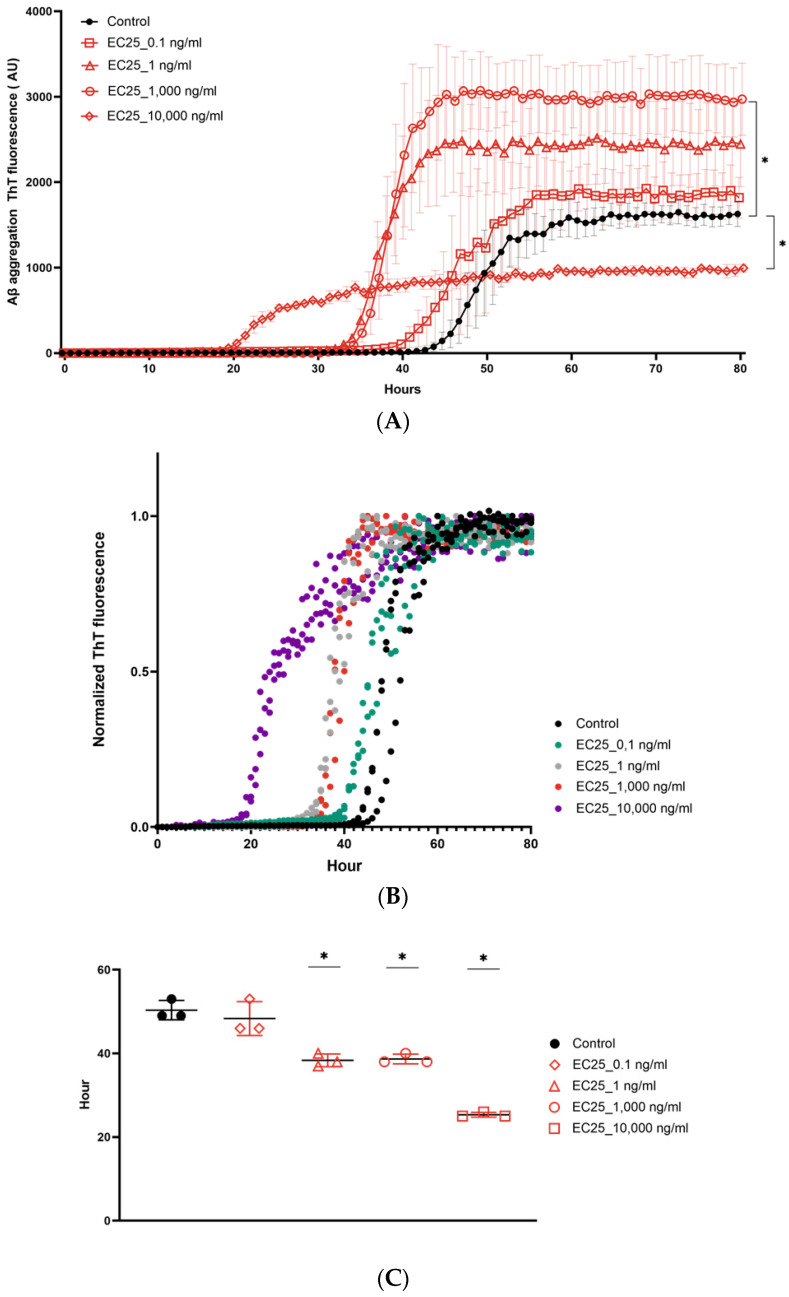

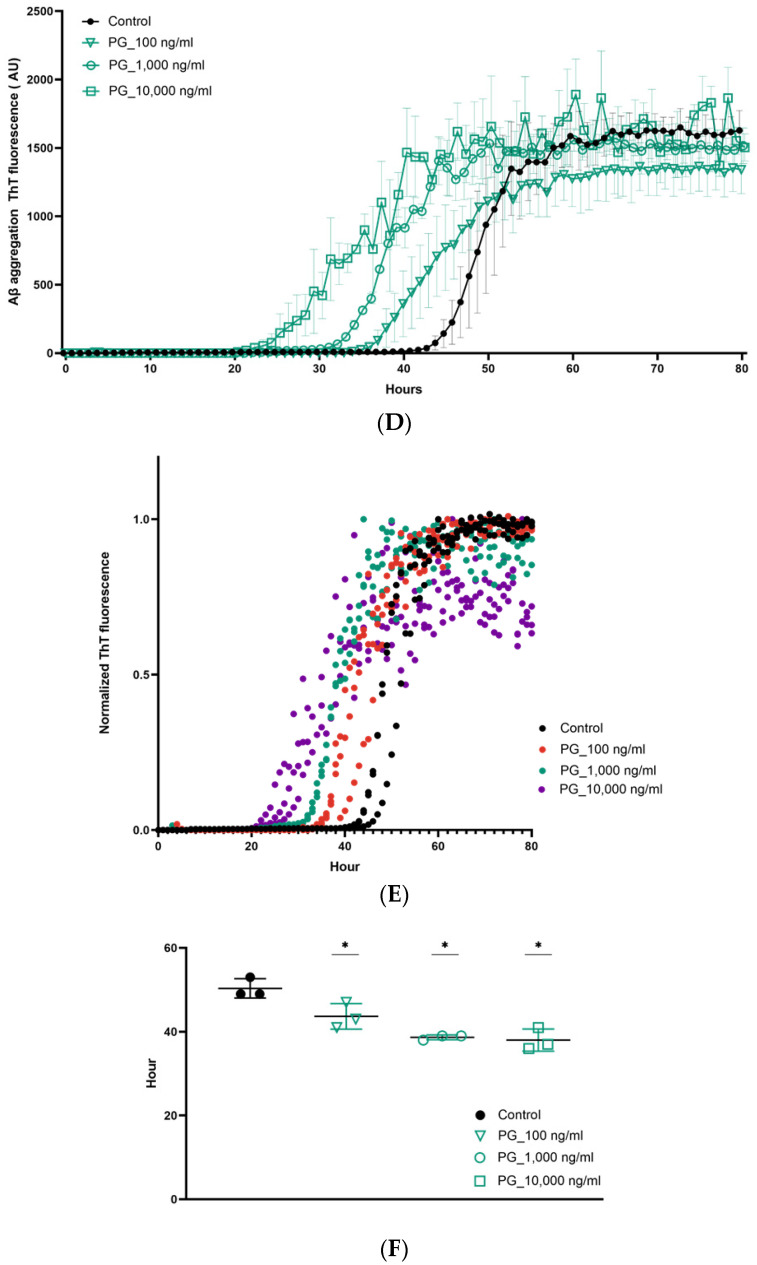
Dose-dependent effect of eDNA on amyloid β (Aβ) aggregation. Dose-dependent effect of DNA from *Escherichia coli* ATCC29522 and *Porphyromonas gingivalis* on Aβ aggregation. Effect of the eDNA of *E. coli* ATCC29522 on (**A**) the aggregation of ThT fluorescence as a function of time, (**B**) normalized ThT fluorescence (relative aggregate concentration) as a function of time (h), and (**C**) t1/2 values of the aggregation. Effect of the eDNA of *P. gingivalis* on (**D**) the aggregation of ThT fluorescence as a function of time, (**E**) normalized ThT fluorescence (relative aggregate concentration) as a function of time (h), and (**F**) t1/2 values of aggregation. For the images (**A**,**C**,**D**,**F**) symbols represent averages and error bars represent the standard deviation of experiments performed in triplicate. * *p* ≤ 0.05; For the images (**B**,**E**) all replicates from three experiments are shown in the plot.

To explain this difference in the aggregation potential of various *E. coli* DNA samples, we analyzed eDNA sizes using an Agilent Bioanalyzer (Figure 2C). Surprisingly, the analyses showed no visible differences between eDNA from different *E. coli* strains consisting of fragments of approximately 50 kb with identical amounts of lower molecular weight DNA. The dependence of Aβ ThT fluorescence on the bacterial DNA concentration was then examined using eDNA from *E. coli* and *P. gingivalis* by a ThT assay (Figure 3A,B). eDNA of *E. coli* ATCC25922 added in concentrations varying from 0.1 to 10,000 ng/mL differentially affected Aβ fibrillation. The highest concentration of eDNA from *E. coli* ATCC25922 at 10,000 ng/mL did not increase ThT fluorescence, whereas lower concentrations of DNA from 0.1 to 1000 ng/mL had a clear dose-dependent effect on Aβ aggregation, with the most pronounced increase in ThT fluorescence of over 2.9-fold following treatment with 1000 ng/mL DNA (Figure 3A). 

By measuring lag-phase and t1/2, we observed that the tested concentrations of eDNA in *E. coli* ATCC25922 had a dose-dependent effect. The highest acceleration of Aβ aggregation was reflected in the shortening of the lag-phase and decrease in the T1/2 found after the addition of the DNA at 10,000 ng/mL (Figure 3B,C). There were fewer pronounced alterations in these parameters after the addition of DNA taken at lower concentrations. eDNA from *P. gingivalis* did not increase ThT fluorescence at any concentration from 100 to 10,000 ng/mL ng/mL. However, eDNA from *P. gingivalis* resulted in a dose-dependent reduction in the lag-phase and t1/2, highlighting its effect on nucleation enhancement, but not on Aβ elongation (Figure 3D–F) [45,46].

## 4. Discussion

The formation and deposition of neurotoxic Aβ aggregates within the brain of patients with AD is a critical pathogenic pathway that causes cognitive decline. Although the molecular basis for Aβ aggregation has been extensively studied, and accumulating evidence supports the notion that a prion-like mechanism is responsible for this process, until now, the exact factor responsible for triggering seeded nucleation remained elusive [14,47]. The suspicion that the seeding factor of AD might arise from microorganisms has been advanced by many groups [48,49]. However, the identity of the microbial component that might trigger prionogenic transformation was unclear. 

In our previous studies, we showed that eDNA from different bacterial species can act as a virulence factor and modify a variety of proteins in different ways, including the prion-like templated aggregation of Tau proteins, highlighting the role of bacterial DNA in “genetic information metabolism” [32,33,50]. In this study, we investigated bacterial eDNA from various Gram-positive and Gram-negative bacteria including oral pathogens associated with AD development, which might trigger Aβ misfolding. It is important to highlight that eDNA of these oral pathogens reaches the CNS via multiple ways. In addition to the general mechanism used by non-oral localized microorganisms to reach the brain from the blood stream through an altered blood–brain barrier, some oral bacteria can invade the brain through cranial nerves (e.g., olfactory or trigeminal nerves) and affect the brain more directly [51,52]. The invasion of the brain by bacteria enables the direct secretion of their extracellular DNA or release under certain conditions, such as prophage activation [35,53]. This results in the release of bacterial DNA directly into the CNS, leading to a high local DNA concentration. 

Using the amyloid-specific fluorescent dye thioflavin-T, for which fluorescence increases in intensity as amyloid beta fibrils aggregate, we found that eDNA from all bacteria studied similarly accelerated the kinetics of Aβ aggregation. This was reflected in the shortened lag-phase and decreased t1/2. However, only eDNA of *E. coli* ATCC 25922 resulted in an increase in maximum ThT fluorescence, whereas the eDNA of other bacteria did not alter this parameter. 

The monitoring of eDNA from different *E. coli* strains surprisingly showed that the catalysis of Aβ aggregation has DNA strain-specific characteristics. Thus, only the eDNA from some of the *E. coli* strains shortened the lag phase, whereas others did not affect it. Moreover, among the *E. coli* eDNA samples that did increase nucleation and shorten the lag-phase, only the eDNA of *E. coli* ATCC25922 potentiated elongation and increased ThT fluorescence intensity at the plateau [54,55]. We suggested that the cause for such a difference in ThT fluorescence could be related to the efficacy of triggering Aβ aggregation and the differences in the sizes of eDNA molecules. Intriguingly, analysis of eDNA molecule size from different *E. coli* strains revealed no association between the eDNA band size of an *E.coli* strain and its effect on Aβ aggregation. When viewed in light of our previous data, these results reveal the same lack of a correlation between bacterial DNA size and its effects on tau misfolding [33]. This suggests that the aggregation capacity of eDNA molecules is not only directly relevant to their length. 

At the same time, it is necessary to highlight that DNA is a polyanionic molecule [56]. Moreover, previous studies have shown that the acceleration of fibril formation and protein misfolding can be triggered by polymers and specifically depends on the polyanionic characteristics of such a molecule [57]. For example, glycosaminoglycans, due to their polyanionic nature and the presence of certain binding sites, function as scaffolds in triggering and enhancing the aggregation of peptide molecules, favoring a β-sheet conformation [58]. Therefore, higher ThT fluorescence following seeding mediated by eDNA of *E. coli* ATCC 25922 might occur through the existence of specific binding sites along the polymer compared with eDNA of other *E. coli* strains even though have the same sizes; however, we would like to emphasize that studying the reasons why DNA triggers Aβ aggregation was not the goal of this study [59].

To study the dose dependence of eDNA of *E. coli* and *P. gingivalis* on Aβ aggregation, we used a wide range of concentrations, starting from 0.1 to 10,000 ng/mL. There are no data on the concentration of bacterial DNA in the brain tissue of CSF of patients with AD. Thus, the concentration of bacterial DNA used was approximated based on the range of CSF DNA concentrations, from 1 ng/mL to 600 ng/mL, observed in patients with different diseases [60,61,62]. 

The addition of *E. coli* ATCC25922 eDNA affected Aβ aggregation in a nonlinear fashion because the highest concentrations of DNA (10,000 ng/mL) decreased ThT fluorescence, whereas exposure to lower concentrations of DNA increased it. The reason for such a discrepancy has not been determined, particularly considering that under the same conditions, eDNA from *E. coli* ATCC25922 from 0.1 to 10,000 ng/mL potentiated nucleation in a linear fashion with a dose-dependent acceleration of t1/2. The unconventional aggregation kinetics observed with 10,000 ng/mL of *E. coli* eDNA could be explained by the fact that DNA at such a high concentration triggers the formation of intermediates that might lead to off-pathway effects on mature fibril formation [63]. Since the level of ThT florescence intensity reflects mature fibrils as they bind to β-sheet-rich structures, the formation of β-sheet-poor aggregates during the elongation phase following the addition of *E. coli* eDNA at 10,000 ng/mL might explain the lower ThT fluorescence, along with faster kinetics [44,55,64,65]. The ability of bacterial DNA to trigger the appearance of intermediate oligomeric species and off-pathway end products is particularly interesting since the presence of these species and products is a more important risk factor for the development of amyloid diseases compared to mature fibrils [63]. Thus, the differential effects of eDNA on protein misfolding, which depends on both the amount of eDNA and type of bacteria from which this eDNA is released, may overlap with recent discoveries of the existence of structurally distinct Aβ polymorphs (strains) that contribute to the variety of clinical phenotypes of AD [66,67,68]. Therefore, in the follow-up studies it would be critical to conduct an electronic or atomic force microscopy analysis to examine the effects of eDNA-induced β-amyloid aggregates on the pathogenesis of AD, and to analyze whether eDNA from different bacterial species in different amounts can induce the formation of different strains of prions.

Notably human DNA was not found to increase the ThT fluorescence of Aβ, as in our previous study when it had no effect on Tau aggregation [33]. However, we are planning to investigate whether other types of human DNA, such as neutrophil extracellular traps known for their distinctive features compared with other DNA types, have different effects on Aβ fibrillation and are going to test this in follow-up studies [69]. Our observation that eDNA from different *E. coli* strains differently affected Aβ, meaning that not all bacterial DNA possesses equal Aβ prionogenic activity, might address the question of why some people with signs of bacterial presence in the brain develop AD and some do not [70]. Indeed, many papers describe the presence of bacteria or their components in CSF and postmortem brains not only of individuals with AD, but also in samples of matched controls [18,41,71]. This raises the unanswered question of why some people who have bacteria or Pathogen-Associated Molecular Pattern (PAMPs) present in their CNS develop neurodegeneration and some do not, since the presence of even a small amount of bacterial PAMPs activates microglia and astrocytes, and considering bacterial eDNA as a strain-specific triggering factor of Aβ aggregation might explain this discrepancy [24,72,73,74,75]. These observations raise an intriguing question that has to be addressed in the follow-up studies: could harboring of some *E. coli* and *P. gingivalis* strains, even in asymptomatic patients with no oral or gastrointestinal diseases, be a potential risk factor for AD.

Moreover, our data outline a new potential mechanism through which distantly localized bacteria, including those localized in the gut, exert effects via the secretion and release of eDNA, which can pass through the blood–brain barrier and could remotely contribute to AD development and aggravate its progression. Our findings are in accordance with the dual-hit hypothesis, which states that the pathological aggregation of prions starts in the enteric nervous system (ENS), which retrogradely spreads via the vagus nerve to the brain [76,77,78,79,80]. Hence, the presence of certain bacterial strains in the gut, in which eDNA can promote protein propagation, should be further studied.

These observations raise the intriguing question of whether bacterial DNA could contribute to the pathogenesis of AD; however, many more future studies are required. Thus, elucidating the precise structural differences in different eDNA types such as the CG/AT ratio, DNA forms, minimal length of DNA to accelerate fibrillation, and time and concentration dependence, as well as experiments showing changes in fibril structure and aggregation using the DNA of strains isolated from the CNS and post-mortem brains of patients with AD are critical for future studies. Overall, our data add another line of evidence that bacterial eDNA as a virulence factor might have additional pathogenic mechanisms through protein aggregation, which could lead to the future translational potential of this discovery.

## Data Availability

Not applicable.
